# „Rasse“ als globaler Datenstrom: Die Hamburger Anthropologie des 20. Jahrhunderts als Ausgangspunkt einer Datengeschichte der Rassifizierung

**DOI:** 10.1007/s00048-023-00370-1

**Published:** 2023-11-29

**Authors:** Philipp Kröger

**Affiliations:** Fakultät I: Philosophische Fakultät, Historisches Seminar, Universität Siegen, Siegen, Deutschland

**Keywords:** Physische Anthropologie, Populationsgenetik, Datengeschichte, Rassifizierung, Physical anthropology, Population genetics, Data history, Racialization

## Abstract

Dieser Aufsatz zeigt anhand der Hamburger Anthropologie des 20. Jahrhunderts, wie historisch spezifische Aufschreibesysteme der anthropologischen Forschung die jeweiligen Vorstellungen davon, was „Rasse“ sei, prägten und diese für den politischen Zugriff operationalisierten. Es werden drei Paradigmen der Geschichte der deutschen Anthropologie – physische Anthropologie, „Erblehre“, Populationsgenetik – untersucht und die Frage gestellt, wie sich die ihnen jeweils zugrunde liegenden Medientechniken – Loseblattsammlung, Kartei, elektronische Datenverarbeitung – auf die Konstruktion und politische Mobilisierung von „Rasse“ auswirkten. Mit einer so konturierten Datengeschichte der Rassifizierung lassen sich die ontologischen Fallstricke jüngerer Debatten um die Kategorie „Rasse“ umgehen.

## Abstract (en)

This article explores anthropological research conducted in Hamburg during the 20th century and demonstrates how historically specific discourse networks (*Aufschreibesysteme*) shaped concepts of race and their subsequent use in politics. To this end, this study examines three paradigms within the history of German anthropology in terms of their underlying inscription technique: physical anthropology/loose-leaf collection, “Erblehre”/card index, and population genetics/electronic data processing. By outlining a data history of racialization, this article avoids the ontological pitfalls of recent debates about the category of race.

Kurz vor der Wende zum zweiten Jahrtausend diskutierte die deutsche Öffentlichkeit in den Spalten überregionaler Zeitungen darüber, „was Rassen sind“. Diesen Titel wählte die *Süddeutsche Zeitung* im Dezember 1996 für einen Leserbrief Rainer Knußmanns (1936–2017), Inhaber des Lehrstuhls für Anthropologie an der Universität Hamburg.^1^ Rund zwei Wochen zuvor war ebenfalls in der *SZ* ein Artikel über die „AG gegen Rassenkunde“ erschienen. Unter diesem Namen kritisierten Studierende den „biologistischen und rassistischen Ansatz“ an Knußmanns Institut. Knußmann verteidigte sich damit, dass die Einteilung von Menschen in „Rassen“ keine rassistische Praxis sei – im Gegenteil: Nur wenn man sage, „was Rassen wirklich sind“, ließe sich Rassist:innen etwas entgegensetzen. Für seine Kritiker:innen hingegen galt es, diese Kategorien „endlich zu demontieren. Denn solange davon ausgegangen wird, daß es ‚Rassen‘ gibt, wird diese Annahme den Rassismus stützen“ (Diedrich & Knigge 1998: 54). Knußmann konterte, indem er auf seine Forschungsergebnisse verwies. Er könne „über genetisch bedingte […] Unterschiede zwischen Menschen nicht hinwegsehen.“^2^ Die Debatte verengte sich auf das „Verhältnis von Natur und Kultur des Menschen“^3^. Zugespitzt lautete die Frage, ob „Rassen“ in der Natur selbst zu finden seien – so Knußmanns Position – oder ob sie ein kulturelles Konstrukt wären. Letztlich blieb die Debatte ergebnislos; die Fronten waren verhärtet. Darin ähnelt sie jüngeren Auseinandersetzungen infolge des Revivals biologistischer Konzeptionen von Gruppenzugehörigkeit durch die hohe Popularität molekulargenetischer Differenzkonstruktionen.

Offensichtlich – darauf verweisen auch Veronika Lipphardt und Jens Niewöhner – bedarf es neuer Wege der Kritik, die zwischen „naturalistischen und soziozentrischen Ansätzen“ operieren (Lipphardt & Niewöhner 2008: 1157). Diese wiederholen nicht die ontologischen Fallstricke obiger Debatten, indem sie fragen, „was Rassen wirklich sind“. Vielmehr analysieren sie – auch im Einklang mit der neueren Nationalismusforschung (etwa Brubaker 2002) –, wie „die Differenzkategorie Rasse selbst in heterogenen Praktiken überhaupt hervorgebracht und wirkmächtig“ wird (Plümecke & Schramm 2022: 180). Ein zentrales Moment in der Konstruktion und damit Herstellung moderner Differenzkategorien, so haben es neuere Studien sowohl zur sogenannten Nationalitätenstatistik (Hansen 2015; Göderle 2016; Kröger 2023) als auch zur anthropologischen Forschung im 19. und 20. Jahrhundert gezeigt (Hanke 2007; Lange 2013; Etzemüller 2015; Germann 2016), waren und sind Praktiken und Techniken der Datenverarbeitung. Letztere schrieben zudem die von ihnen hergestellten Differenzkategorien fest und operationalisierten sie für den politischen Zugriff. Von diesen Studien inspiriert, lenkt der vorliegende Aufsatz den Blick auf die Aufschreibesysteme der anthropologischen Forschung im 20. Jahrhundert und folgt den von ihnen produzierten Datenströmen. Diese, so eine erste These, stellten her, was als Natur ausgelegt wurde: Die Techniken und Praktiken der Datenproduktion, -speicherung und -zirkulation naturalisierten Differenzkonstruktionen und verankerten sie im Materiellen. Der Fokus richtet sich also auf die nach wie vor vernachlässigte Materialität und technische Infrastruktur von Klassifikationssystemen (Bowker & Star 2000: 33–50).

Auf die Relevanz von Techniken und Praktiken der Verdatung innerhalb der Wissensproduktion verwies bereits Bruno Latour in seinen frühen Laborstudien. So gerieten insbesondere die „inscription devices“ und die Visualisierungspraktiken datengetriebener Forschung in den Blick – dabei lässt sich die Bedeutung von Inskriptionen auch jenseits des Labors ausmachen und verallgemeinern (Latour 1983: 161; Latour 2006). In jüngerer Zeit entstandene Ansätze können unter dem Begriff „data histories“ subsumiert werden (Aronova et al. 2017). Auch im Anschluss an Latour betonen diese, dass Daten nicht als Gegebenes ein unvermitteltes Abbild der Wirklichkeit repräsentieren. Vielmehr bilden sie als Gemachtes kein passives Element der Wissensproduktion. Sie waren und sind, so drückt es Theodore Porter in seiner Studie zur frühen Vererbungsforschung aus, nicht selten ihr „prime mover“ (Porter 2018: 33). Insofern gilt es, einen Blick unter die Oberfläche vermeintlicher Evidenz von Datensätzen – „at the machinery by which these fine compilations have been generated“ (Chadarevian & Porter 2018: 553) – zu werfen. Dabei ist für diesen Aufsatz die Frage zentral, wie Medientechniken die Produktion von Wissen beeinflussen. So zeigt etwa Christine von Oertzen anhand des preußischen Zensus im 19. Jahrhundert, wie die Einführung neuer Papiertechnologien – zunächst Zählblättchen, ab 1871 Zählkarten – nicht nur neue Praktiken der Datenerhebung und -auswertung, sondern damit vor allem neues Wissen über die Bevölkerung hervorbrachte (Oertzen 2017: insb. 415–419).

Daraus ergibt sich im Hinblick auf die hier untersuchte anthropologische Forschung des 20. Jahrhunderts eine zweite These. Die von ihr hervorgebrachten Konstruktionen und Vorstellungen von menschlicher Differenz saßen, so die Annahme, Medientechniken auf und operierten innerhalb spezifischer Aufschreibesysteme. Letztere bezeichnet Friedrich Kittler als das „Netzwerk von Techniken und Institutionen […], die einer gegeben Kultur die Adressierung, Speicherung und Verarbeitung relevanter Daten erlauben“ (Kittler 1995: 519). Einerseits kann darüber in Anlehnung an Cornelius Borck und Armin Schäfer verfolgt werden, wie die „Techniken des Notierens, Beobachtens, Schreibens und Verarbeitens“ die beforschten Objekte hervorbrachten, da sie „mittels dieser Verfahren zuallererst ihre Konturen gewannen.“ (Borck & Schäfer 2015: 24) Andererseits präfigurieren Aufschreibesysteme Diskurse: Die Diskursanalyse wird „vom (textorientierten) Kopf auf ihre (medientechnischen) Füße gestellt“ (Winthrop-Young 2005: 108). Damit ist nicht gemeint, dass Medientechniken von sich aus die Objekte hervorbringen, die sie in Daten abbilden, sondern dass die Frage Foucaults – „[W]ie kommt es, daß eine bestimmte Aussage erschienen ist und keine andere an ihrer Stelle?“ (Foucault 1981: 42) – auch bedingt ist durch das ihnen zugrunde liegende Aufschreibesystem. Anders ausgedrückt: Die Praktiken und Techniken der Verdatung brachten nicht nur hervor, was sie als Repräsentation einer vorgelagerten Wirklichkeit ausgaben; sie schrieben sich auch maßgeblich in die Kategorien menschlicher Differenz ein.

Zeigen lässt sich dies exemplarisch anhand der Hamburger Anthropologie des 20. Jahrhunderts zeigen – von der Gründung der Anthropologischen Abteilung im Hamburger Völkerkundemuseum im Jahr 1906 bis zur Emeritierung Knußmanns im Jahr 1998. Anhand dieses zeitlichen Längsschnitts wird die Geschichte der deutschen Anthropologie mittels dreier Paradigmen untersucht. Diese Paradigmen – physische Anthropologie, „Erblehre“ und Populationsgenetik – bilden die Struktur des vorliegenden Aufsatzes. Er gliedert sich somit in drei Kapitel: zur Hamburger Südsee-Expedition in den Jahren 1908 bis 1910, zur Forschung des Anthropologen Walter Scheidt (1895–1976) in der Zwischenkriegszeit sowie zu Knußmann ab den 1970er Jahren. Die damit einhergehenden Paradigmen waren, so die Annahme, mit einer jeweils spezifischen Medientechnik verknüpft: Loseblattsammlung, Kartei, elektronische Datenverarbeitung. Wie wirkten sich diese, so fragen die Kapitel, auf die Konstruktion und politische Mobilisierung von „Rasse“ aus?

## „Rasse“ als Loseblattsammlung – die Hamburger Südsee-Expedition im Zeichen der physischen Anthropologie

Im Anschluss an ein Referat über das „Inzuchts- und Bastardisierungsproblem beim Menschen“, das Eugen Fischer (1874–1967) beim Kongress der Deutschen Anthropologischen Gesellschaft im Jahr 1911 hielt, meldete sich der Ethnologe Augustin Krämer (1865–1941) zu Wort. Krämer, einer der Leiter der Hamburger Südsee-Expedition (SSE), verwies auf die dort vorgenommenen „umfangreiche[n] anthropologische[n] Untersuchungen“. Sie könnten – so Krämers Überlegung – Fischers Thesen stützen. Die „Ergebnisse der Messungen“ lagen allerdings noch nicht vor (Fischer 1911: 108). Zwischen 1908 und 1910 hatten Hamburger Wissenschaftler in den vom Deutschen Reich annektierten Regionen des heutigen Papua-Neuguineas sowie auf der Inselgruppe der Karolinen Hunderte Menschen vermessen sowie eine Vielzahl an Skeletten, Schädeln und Knochen gesammelt.^4^ Fischer präsentierte beim Kongress Daten, die er im Jahr 1908, jedoch in einer anderen Kolonie – Deutsch-Südwestafrika – erhoben hatte. Im Vergleich zur SSE hatte Fischer deutlich weniger Menschen vermessen und keine Körperteile sammeln können. Während jedoch die von Krämer groß angekündigten Ergebnisse der SSE nur in sehr kleinem Umfang erschienen, publizierte Fischer bald eine vielzitierte Studie (Fischer 1913), die zentral werden sollte für den Paradigmenwechsel von der physischen Anthropologie zur sogenannten „Erblehre“.

Eine Ursache für diesen Paradigmenwechsel findet sich in den zugrunde liegenden Datenpraktiken. Fischer betonte, dass er die von ihm untersuchten Menschen „aktenmäßig“ kenne. Er meinte damit „Stammbaum und Ahnentafeln“ (Fischer 1911: 107), die er angelegt hatte. Mit diesem genealogischen Wissen wollte Fischer ein zentrales Problem der um 1900 kriselnden Anthropologie lösen: die sogenannte „Rassenmischung“. Die Hamburger Expedition operierte indes innerhalb der Datenpraktiken der physischen Anthropologie. Diese gilt es zunächst anhand des Materials der SSE zu erkunden und zu fragen, wie darüber „Rasse“ hergestellt wurde, aber auch, warum sie in die Krise geriet und abgelöst wurde.

Eine zentrale Aufgabe der SSE bestand in der „Abgrenzung der Völker“, die unter dem Begriff „Melanesier“ firmierten, von jenen, die „Mikronesier“ oder auch „Polynesier“ genannt wurden (Fischer 1981: 23). Neben anthropologischen Aufnahmen sollten dafür vor allem völkerkundliche Forschungen zur indigenen Bevölkerung herangezogen werden. Dahinter stand auch ein ökonomisches Interesse. Die Hamburgische Wissenschaftliche Stiftung finanzierte die Expedition mit Spenden der Kaufmannschaft – die Südsee galt als kolonialer Expansionsraum Hamburger Handelshäuser und Firmen; manche waren dort bereits aktiv (Laukötter 2007: 169). Georg Thilenius (1868–1937), Direktor des Völkerkundemuseums und Initiator der Expedition, sprach daher von einer „praktischen Aufgabe“ (zit. n. Fischer 1981: 38): Das akkumulierte Wissen sollte sich in der Akkumulation von Kapital niederschlagen. So galt es etwa die Ursachen eines wahrgenommenen Bevölkerungsrückgangs zu klären, der die Zahl potenziell ausbeutbarer Arbeitskräfte minimierte. Zudem ließ im zeitgenössischen Denken die Vermessung der indigenen Bevölkerung auch Rückschlüsse auf ihre Einsatzmöglichkeiten zu. Etwa verknüpfte der am Völkerkundemuseum tätige Ethnologe Paul Hambruch (1882–1933) seine Beobachtungen über die Körperbeschaffenheit von Menschen mit Aussagen über angenommene „Arbeitsleistungen“ (Hambruch 1914: 70). Offensichtlich, so diese Logik, ermöglichte die Einteilung der indigenen Bevölkerung in Differenzkategorien zugleich die Koordination der kolonialen Menschenökonomie. „Die Lösung der Arbeiterfrage“, so drückte es Thilenius aus, setze „die genaue Kenntnis der Bevölkerung voraus“ (zit. n. Fischer 1981: 38). „Der Anthropologe“ hätte „möglichst viele Individuen zu messen und körperlich zu untersuchen“ (zit. n. Fischer 1981: 93).

Diese Massenstatistik entsprach dem Programm der physischen Anthropologie, die eine „Wissenschaft des Sammelns und Katalogisierens“ war (Weingart et al. 1988: 355). Anhand der umfänglichen Vermessung von Menschen sowie ihrer sterblichen Überreste wurde versucht, auf „Typen“ beziehungsweise „Rassen“ zu schließen. Die Konzentration auf morphologische Differenz hatte sich im Verlauf des 19. Jahrhunderts verfestigt. Die sich zuvor semantisch überlagernden Begriffe von Volk und „Rasse“ differenzierten sich dabei nach und nach aus. Den Hintergrund dafür bildete unter anderem eine groß angelegte Studie Rudolf Virchows (1821–1902) an Schulkindern in den 1870er und 80er Jahren. Sie hatte gezeigt, dass „Rasse“ nicht deckungsgleich mit dem sei, was unter Volk oder auch Nation verstanden wurde (Massin 1996: 100). Die anthropologischen Vermessungsmethoden, -techniken und Instrumente, die im Übrigen bereits seit dem 18. Jahrhundert Anwendung fanden (Gould 1988), wurden um 1900 weitestgehend standardisiert. Im deutschen Sprachraum schrieb der Anthropologe Rudolf Martin (1864–1925) diese für die kommenden Jahrzehnte in seinem 1914 publizierten *Lehrbuch der Anthropologie* fest (Eickstedt 1963 [1940]: 442–443). „Rasse“ formierte sich dabei vor dem Hintergrund ihrer datengestützten Übersetzbarkeit als eine Gruppe von Menschen, die „eine Summe von Merkmalen gemeinsam haben und sich durch eben diese bestimmte Merkmalskombination (Merkmalskomplex)“ von anderen Gruppen unterschieden (Martin 1914: 7). Es bedurfte also zunächst einer Vielzahl von Daten über die körperliche Beschaffenheit von Menschen, um die Kategorie der „Rasse“ herzustellen.

Zentrales Medium der physischen Anthropologie war die Loseblattsammlung: die Erfassung von Menschen über „Individualbeobachtungsblätter“ (Martin 1914: 61). Hunderte dieser standardisierten Bögen dienten auch während der SSE dazu, die indigene Bevölkerung in Daten zu übersetzen.^5^ Zuständig dafür war neben Paul Hambruch vor allem Otto Reche (1879–1966), Leiter der 1906 begründeten Anthropologischen Abteilung des Völkerkundemuseums und späterer NS-Rassenforscher (Geisenhainer 2002). Bei einer Durchsicht ihrer Expeditionstagebücher^6^ zeigt sich, dass über die Anlage von Beobachtungsblättern das Sammeln und Katalogisieren tatsächlich die zentrale Praxis war. Weder Reche noch Hambruch trafen eine Auswahl ihrer Untersuchungsobjekte, sondern vermaßen, wann immer sie konnten – beziehungsweise wenn sich die indigene Bevölkerung, teils unter Androhung von Gewalt, dazu bewegen ließ. Anders ausgedrückt: Die Medientechnik der individuellen Beobachtungsblätter, die untereinander zunächst keinen Zusammenhang bildeten, war blickbildend und bestimmte das Denken sowie die Forschungspraxis der Wissenschaftler. Sie bedingte eine mehr oder minder zufällige Auswahl an Untersuchungsobjekten, die erst im Nachhinein – bei der Auswertung – in Beziehung zueinander gesetzt wurden.

**Figure 1:**
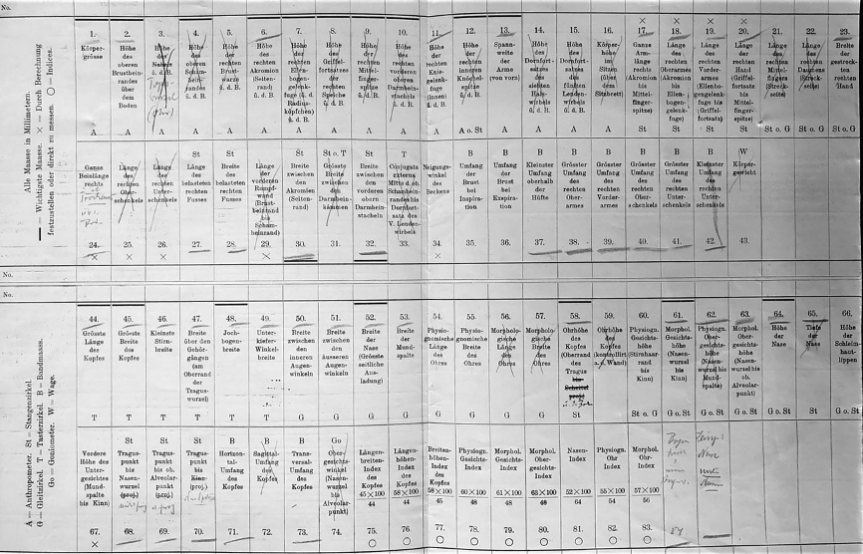

Technik und Methode der Datenaufnahme diktierten die Beobachtungsblätter hingegen strikt. Die „vieljährige […] Erfahrung“ führender Anthropologen war in sie eingeschrieben (Martin 1914: 62). Jedes Blatt hatte ein Format von 40 × 26,5 Zentimeter, wurde in der Mitte gefaltet und bestand somit aus insgesamt vier Seiten. Zentral waren die beiden mittleren, die zur Aufnahme anthropometrischer Daten dienten. Die Blätter waren so angelegt, dass die Messungen den Instrumenten entsprechend nach einer bestimmten Reihenfolge ausgeführt wurden. Sie boten Platz für 74 einzelne Messungen – von der Körpergröße über die Beinlänge bis zum Kopfumfang – sowie neun aus diesen Daten errechenbare Indizes. Es bedurfte dafür sieben verschiedener Instrumente: Anthropometer, Stangenzirkel, Gleit- und Tasterzirkel, Bandmass, Goniometer sowie einer Waage. Indes waren nicht alle Messungen und Instrumente notwendig. Auf einem „Probeblatt“ hatte Reche für die Expedition 44 Maße rot markiert (Abb. 1) –7 aber selbst diese ergaben für jedes erfasste Individuum eine Vielzahl an Daten.

„Rasse“ und damit die Einteilung der vermessenen Menschen in Gruppen entstand erst in der Auswertung des Materials, die im Hamburger Völkerkundemuseum erfolgte. Generell gestaltete sich die Suche nach „Typen“ und „Rassen“ in der physischen Anthropologie als eine komplexe Verschaltung von Datensätzen. Indizes wurden berechnet, Mittelwerte und Korrelationskoeffizienten gebildet; anschließend visualisierten Grafen und Diagramme die Ergebnisse (Martin 1914: 63–103; Hanke 2007). Die Übersetzung von Menschen in Daten war somit einerseits Bedingung für die Konstruktion von „Rassen“, andererseits begrenzte sie die Möglichkeiten ihrer Herstellung.

**Figure 2:**
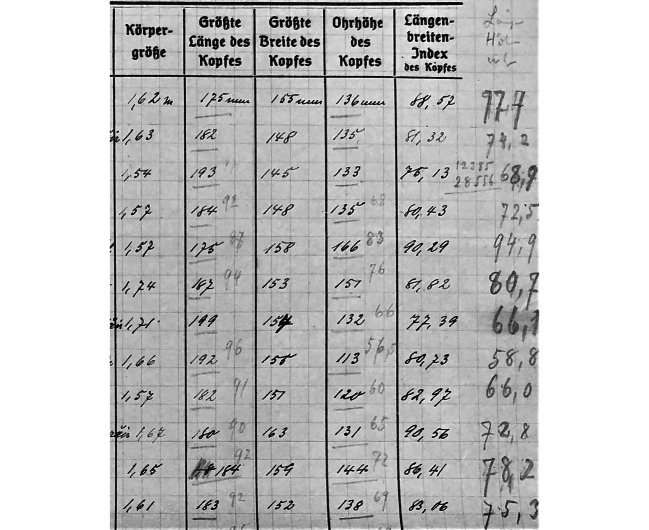

Zunächst transformierte die Übersetzung die Heterogenität der vermessenen Menschen in einen standardisierten Datensatz, der diese vergleich- und berechenbar machte. Das Einschreiben der Vermessenen in ein Beobachtungsblatt war der erste Schritt in einer langen Kette von Übersetzungen, die aus dem Materiellen einen diskursfähigen Gegenstand produzierte. Die Menschen, die Reche und Hambruch vermaßen, ließen sich als solche nicht seriell verbinden – in Messbögen übersetzt hingegen schon. Es ist jedoch mit Latour, auf den diese Überlegungen zurückzuführen sind, zu betonen, dass jeder Schritt dieser Kette – von der Datenaufnahme während der Expedition bis zu deren Auswertung in Hamburg – eine Transformation darstellt. Die Daten bildeten kein einfaches Abbild einer vorgelagerten Wirklichkeit, sondern produzieren etwas Neues (Latour 1999: 69–73). Die Datenaufnahme sowie -speicherung ermöglichte somit die Überwindung von Zeit und Raum. Alle „Momente in der Zeit und alle Orte im Raum“ können über ihre Verdatung „in einem anderen Raum und in einer anderen Zeit gesammelt werden“ (Latour 2006: 286). Die Sammlung gleichförmiger Daten an einem Ort ermöglichte wiederum ihre Kombination. Paul Hambruch etwa hatte die einzelnen Messblätter, die er auf der Insel Palau angefertigt hatte, zur Auswertung der Daten in eine Tabelle (Abb. 2) übertragen.^8^ Erst solche Tabellen stellten eine Beziehung der vermessenen Menschen untereinander her und dienten als Grundlage weiterer Berechnungen. Die darüber ermöglichte Ableitung von „Typen“ oder auch „Rassen“ war nur auf dem Papier möglich.

Die bei der Auswertung des primären Datensatzes gewonnenen Ergebnisse ließen sich wiederum mit anderen Studien und Datensätzen verschalten. Hambruch etwa verglich die Resultate, die er anhand seiner Messungen auf Nauru erzielte, mit Schädelmessungen, die vor der Jahrhundertwende erfolgt waren (Hambruch 1914: 77–78). Reche kombinierte für eine theoretische Arbeit über den Schädelindex die von ihm gesammelten und vermessenen Schädel der SSE mit einer Reihe anderer Datensätze (Reche 1911). „Rasse“ entstand demzufolge innerhalb eines globalen Datenstroms: Die Beobachtungsblätter übersetzten Menschen in Datensätze, die sich kombinieren ließen. Darüber entstand in einem mehrstufigen Verfahren Ähnlichkeit und Differenz.

Diese Datenpraktiken, die den Kern der physischen Anthropologie bildeten, waren zugleich ihre Schwäche und um 1900 geriet sie in die Krise (Massin 1996: 106–114). Trotz zunehmender Präzision der Messtechnik und einer größer werdenden Datenmenge konnte hinsichtlich der zentralen Aufgabe – der Identifikation von „Rassen“ – kein Durchbruch erzielt werden. Waren die in Mittelwerten gefundenen „Rassen“ tatsächlich „Rassen“, oder waren sie aus Mischungen ursprünglicher „Rassen“ entstanden? Paul Hambruch meinte auf der Insel Nauru zwei „Bevölkerungselemente“ zu erkennen. Das „Vorhandensein von mindestens zwei Typen“ sei „wahrscheinlich“ – allerdings seien die „Unterschiede verwischt und die Übergänge so mannigfaltig“, dass sie sich nicht fixieren ließen. „So präzise“ wollte Hambruch seine „Beobachtungen nicht festlegen.“ (Hambruch 1914: 72) Otto Reche hatte zunächst in einem kurzen Artikel über seine Studien am „Kaiserin-Augusta-Fluss“ (heute Sepik in Papua-Neuguinea) „drei deutlich zu trennende Typen eventuell Rassen“ erkannt (Reche 1910: 286). Wenige Jahre später sprach er bereits von fünf. Es war Reche zwar möglich, „Typen“ zu bilden, jedoch sei die „Bevölkerung in der Hauptsache als ein Kreuzungsprodukt“ dieser „Typen“ beziehungsweise „Rassen“ anzusehen (Reche 1913: 57). Einerseits ließen sich über die gesammelten Daten „Rassen“ überhaupt erst konstruieren, andererseits löste diese „Mathematisierung“, so Christine Hanke, das „Konzept ‚Rasse‘ auf“ (Hanke 2007: 61).

Paul Hambruch kündigte entsprechend an, dass er seine „Messungen, unter Anwendung der von E. Fischer bei den Rehobother Bastards vorgezeichneten neuen Wegen und strenger Kritik“ durcharbeiten wolle (Hambruch 1914: 72). Was Hambruch zu diesem Zeitpunkt offenbar nicht wusste: Die in Individualerhebungsbögen gespeicherten Daten waren nicht kompatibel mit jenen „neuen Wegen“, die Eugen Fischer beschritt. Otto Reche hörte im August 1913 einen Vortrag Fischers und notierte, dass „eine Reihe von Rassenmerkmalen“ wie Haarform, Körpergröße und Hautfarbe für die „Beurteilung der Verwandtschaft verschiedener Typen nicht verwendbar sind.“^9^ Es waren nämlich nicht die „Massenuntersuchungen, nicht ‚Typen‘, Mittelwerte und Variationsbreiten“, so Fischer, die bei der Suche nach „Rassen“ interessierten, sondern die „Familienstämme“. In der „Familienanthropologie“ lag die „Zukunft wenigstens des Rasseteils der Anthropologie“ (Fischer 1913: 2).

Um 1900 hatte ein Prozess stattgefunden, den Hans-Jörg Rheinberger und Staffan Müller-Wille als „Disziplinierung der Vererbung“ beschreiben (Rheinberger & Müller-Wille 2009: 169–208). Die Regeln, die Gregor Mendel (1822–1884) in der Mitte des 19. Jahrhunderts entdeckt und als Spezialfälle interpretiert hatte, ließen sich nun als allgemeines Gesetz der Vererbung formulieren. Es kam zur „Etablierung eines Experimentalsystems“, in dem sich Vererbung zu „einem epistemischen *Objekt* verdichtete.“ (Rheinberger & Müller-Wille 2009: 183) Mithin entstanden die Begriffe Gen und Genetik. Auch hier waren Datenpraktiken Movens der Entwicklung. In der Tier- und Pflanzenzucht waren im 19. Jahrhundert „neue Verzeichnis- und Kalkulationsformen“ entstanden (Rheinberger & Müller-Wille 2009: 178). Statistische und genealogische Daten wurden in Zuchtregistern erfasst, die in der wissenschaftlichen Züchtungsforschung mündeten – ein zentraler Ort des Mendelismus um 1900. Im 19. Jahrhundert hatte sich zudem über das verstärkte Interesse an genealogischer Datenerhebung und ihrer Visualisierung in Stammbäumen ein neues Verständnis von Verwandtschaft etabliert, das starken Einfluss auf den Vererbungsdiskurs, aber auch auf Vorstellungen von „Rasse“ ausübte (Müller-Wille 2023; Rheinberger & Müller-Wille 2009: 155–168). Auch in der psychiatrischen Forschung in Deutschland lässt sich um 1900 die Abkehr von der Massenstatistik und die Hinwendung zu genealogischen Aufzeichnungen ausmachen (Gausemeier 2008: 145; Porter 2018: 281–311).

In Fischers eingangs erwähnter Studie bündelten sich diese Entwicklungen. Aus zeitgenössischer Perspektive erbrachte er den Nachweis, demzufolge sich morphologische Merkmale des Menschen gemäß den Mendelschen Regeln vererben würden. Diese bislang fehlenden „Kenntnisse […] über die gesetzmäßigen Vorgänge bei Rassenmischung“ sollten „zu einer wirklichen Lösung der Frage der Rassenmischungen“ beitragen und damit ein zentrales Problem der physischen Anthropologie adressieren (Fischer 1913: 1). Die entstehende Genetik verlangte als Experimentalwissenschaft jedoch nach einem Labor. Während „Botaniker und Zoologen“, so Fischer, „mit vielen Tausenden von beobachteten und genealogisch genau bestimmten Tieren und Millionen solcher Pflanzen experimentieren“, müsse der Anthropologe beobachten, „wo die Natur und des Menschen unberechenbare Laune ihm freiwillig ein Experiment vormachen“ (Fischer 1913: 2). Indes musste auch dieses wiederum über Daten rekonstruierbar sein: „Das größte Rassenkreuzungsexperiment am Menschen – Weißer und N[…] in Amerika – ist wissenschaftlich ungenutzt abgelaufen“; die „Aszendenz“ der schwarzen Bevölkerung sei nicht zugänglich (Fischer 1913: 2). Im Gegensatz dazu ließen sich für sein Untersuchungsobjekt, die sogenannten „Rehobother Bastards“, die Stammbäume aus den Akten – Taufregister, Familienregister und -listen der Distriktregierung (Fischer 1913: 23, 47) – erstellen, was ihm aus seiner Perspektive den Zugriff auf Vererbungsmechanismen ermöglichte.

Das Aufschreibesystem der Loseblattsammlung führte dabei jedoch in eine Sackgasse. Die allein auf die Physis zielende Kompilation von Einzelaufnahmen, die erst anschließend serialisiert wurde, gelangte an ihre Grenzen. Erstens bedingte die Anlage individueller Erhebungsbögen eine mehr oder minder willkürliche Auswahl der vermessenen Menschen, die erst in der Auswertung der Daten in Beziehung zueinander gesetzt und darüber zu „Rassen“ oder auch „Typen“ gruppiert wurden. Die darin immer wieder zum Vorschein tretenden Widersprüche fixierten also die gedachten Kategorien nicht, sondern lösten sie vielmehr auf. Damit war auch die ihnen zugedachte Funktion in der Kolonialverwaltung und Menschenökonomie nicht umsetzbar. „Rasse“ ließ sich nur bedingt in materielle Herrschaft überführen, wenn sie sich nicht in Daten übersetzen ließ. Zweitens deuteten die Wissenschaftler diese in ihrem Datenmaterial auftretenden Widersprüche zumeist als sogenannte „Rassenmischungen“. Letztere verwiesen jedoch auf eine den Differenzkategorien zugedachte Dynamik, die das Aufschreibesystem der physischen Anthropologie nicht auffangen konnte. So galten die von ihr berechneten „Typen“ und „Rassen“ als weitestgehend unveränderbar und überzeitlich (Lange 2013: 187). Diese Statik spiegelte sich in den Aufnahmebögen wider. Sie bedingten eine Konzeption menschlicher Körper, die – wie die über ihre Vermessung berechneten „Rassen“ – jenseits von Zeit und Raum existierten, während Fischer an historisch gewordene Vererbungslinien dachte.

Im Jahr 1928 fasste Fischer, nun Direktor des Kaiser Wilhelm-Instituts für Anthropologie, menschliche Erblehre und Eugenik, das Forschungsprogramm der „Erblehre“ in einem Antrag an die Notgemeinschaft der deutschen Wissenschaft noch einmal zusammen. Die Anthropologie solle die „Bevölkerung in ihrem genealogischen und historischen Zusammenhang untersuchen“ und damit „eine wirkliche Anthropologie, nicht anthropologische Einzeldaten an willkürlich bezw. zufällig herausgezogenen Einzelindividuen erhalten.“^10^ Als Vorbild dafür verwies Fischer auf eine Studie über die Elbinsel Finkenwerder des Anthropologen Walter Scheidt, der im Jahr 1924 die Nachfolge Reches im Hamburger Völkerkundemuseum angetreten hatte. Statt durch großformatige Individualerhebungsblätter war seine Forschung von der Medientechnik der Kartei bestimmt.

## „Rasse“ aus dem Karteikasten – Walther Scheidts Studien zur „Erblehre“ und die nationalsozialistische Rassenpolitik

Für Walter Scheidt war „Rasse“ weder ein abstrakter noch ein metaphysischer Begriff. „Sogenannte Rassensysteme und Bilderbücher“ waren für ihn keine geeigneten Mittel seiner Repräsentation. Man solle die „Volksgemeinschaft“ nicht in „Wolkenkuckucksheim – oder in leeren Worten suchen“, so Scheidt im August 1933 in der *Kölnischen Zeitung* (Scheidt 1933: 2–3). „Rasse“ war damit ebenso wenig in den Messreihen der physischen Anthropologie zu finden, die er im Anschluss an Eugen Fischer kritisierte. „Volk und Rasse“ – so auch der Titel einer Zeitschrift, die Scheidt 1925 begründete – waren für ihn konkrete biologische Entitäten: „Das Dasein einer Rasse ist wirklich real“ (Scheidt 1925: 341). Um sie zu erfassen, hatten Scheidt und seine Mitarbeiter:innen in der „rassenkundlichen Abteilung“ des Völkerkundemuseums, so ihr neuer Name (ab Oktober 1933 „Rassenbiologisches Institut“ an der Universität Hamburg)^11^, eine „rassenbiologische“ Kartei aufgebaut. In massiven Schubschränken waren im Jahr 1932 bereits rund 500.000 Karten untergebracht. Sie speicherten Daten von „ca. 250.000 Personen“. Auf dieser Basis dachte Scheidt gar über eine „bevölkerungsbiologische Zentralkartei des deutschen Volkes“ nach. In dieser wäre „wirklich der ganze Volkskörper“ vertreten und „zur Auskunft an einem Ort anwesend“ (Scheidt 1932: 565–566).

In den 1920er und 1930er Jahren nahm Walter Scheidt eine „Spitzenposition“ (Roth 1984: 116) in der deutschen Anthropologie ein und übte großen Einfluss auf sie aus (Gausemeier 2008: 161). Nicht nur wurden seine „rassenkundlichen“ Studien Vorbild für besagten Forschungsantrag Eugen Fischers; vor allem wurde die dabei entwickelte Karteitechnik zu einem wichtigen Medium der „Erblehre“ sowie der NS-Rassenpolitik. Indes geriet Scheidt 1933 ins wissenschaftliche und politische Abseits. Seine Äußerungen über „Bilderbücher“ richteten sich auch gegen Hans F. K. Günthers (1891–1968) populäre Schriften zur „nordischen Rassenlehre“, die eine zentrale Stellung in nationalsozialistischen Vorstellungswelten einnahm (Essner 2002: 61–75). Walter Groß (1904–1945) vom Aufklärungsamt für Bevölkerungspolitik und Rassenpflege, dem Vorläufer des Rassenpolitischen Amtes der NSDAP (RPA), drohte Scheidt im Jahr 1933 mit Forschungs- und Publikationsverbot.^12^ In der Folge verlor Scheidt die Förderung für sein wichtigstes Forschungsprojekt – die *Deutsche Rassenkunde* (so auch der Name der daraus entstandenen Schriftenreihe). Vier Jahre später unterschrieb Groß jedoch gemeinsam mit „Reichsbauernführer“ Walther Darré (1895–1953) sowie dem Leiter des Nationalsozialistischen Lehrerbunds eine Vereinbarung zur Totalerfassung des deutschen Volkes „für rassenpolitische und sippenpflegerische Aufgaben“^13^. Zentral dafür war jene von Scheidt erdachte und von seinem Mitarbeiter Wilhelm Klenck (1890–1959) verfeinerte Karteitechnik (Klenck & Kopf 1938).

Die Anfänge dieser Technik finden sich in einer Studie Scheidts auf Finkenwerder – in den 1920er Jahren noch eine Insel vor den Toren Hamburgs. Scheidt hatte sich, bevor er nach Hamburg kam, bei Rudolf Martin in München habilitiert. Die Abkehr von der physischen Anthropologie und die Hinwendung zur „Erblehre“ – das entsprach einer allgemeinen Tendenz innerhalb der deutschen Anthropologie (Proctor 1988: 147) – war jedoch bereits zu Beginn der 1920er Jahre erfolgt (Scheidt 1954: 3). Erste Ansätze empirischer Forschung in München setzte Scheidt in Hamburg fort. Dort hatte der Direktor des Altonaer Museums, Otto Lehmann (1865–1951), bereits im Jahr 1918 Desiderata der „biologisch-statistische[n] Forschung“ in der Feststellung des „Genotypus“ einzelner deutscher „Stämme“ erkannt (Lehmann 1918: 503). Über Lehmann erhielt Scheidt nicht nur finanzielle und ideelle Unterstützung, sondern vor allem Zugang zu regionalen Heimatforschern, so auch auf der Elbinsel Finkenwerder.^14^ Im Jahr 1925 führten der Hamburger Anthropologe und seine Mitarbeiter:innen bereits in mehreren ländlichen Regionen, insbesondere in Niedersachsen, „rassenkundliche[…] Erhebungen“ durch.^15^

Der Fokus auf rurale Gemeinden und die Kooperation mit Heimatvereinen und Genealogen entsprang Scheidts Verständnis von „Rasse“. Diese war für ihn ein „innerhalb der Art ausgelesener Eigenschaftskomplex“. Damit synthetisierte Scheidt das, was der Rassenhygieniker Alfred Ploetz (1860–1940) als „Vitalrasse“ bezeichnete – also „Etwas in der Erbmasse Liegendes“ – mit jenem typisierenden Konzept, das „Systemrasse“ genannt wurde: „die Ähnlichkeit einer Menschengruppe […], denn Auslese führt immer zu relativer Häufung“ (Scheidt 1925: 331–332). Diese Auslese von Merkmalen war jedoch über die bisherigen anthropologischen Datenpraktiken nicht zugänglich. So wie etwa in der psychiatrischen Forschung versucht wurde, über genealogische Daten mittelbar der „inneren Mechanik der Vererbung beim Menschen ein Stück weit auf die Spur zu kommen“, so wandte sich auch Scheidt der Genealogie zu. Sie erlaubte es, „historische Ereignisse rückblickend so zu analysieren, als habe es sich um Experimente gehandelt“ (Rheinberger & Müller-Wille 2009: 166). Anders ausgedrückt: Erst wenn die verwandtschaftlichen Beziehungen einer Menschengruppe vermeintlich eindeutig geklärt waren, konnte über eine anschließende Vermessung ihrer Körper auf jenen Prozess geschlossen werden, den Scheidt als Auslese von Eigenschaftskomplexen und damit als „Rasse“ bezeichnete.

Scheidts Forschungsprogramm basierte daher zunächst auf einem genealogischen Screening der Bevölkerung. Dabei bildeten Kirchenbücher den basalen Datensatz. Über diesen, so Scheidts Annahme, ließen sich „diejenigen altansässigen Teile der Bevölkerung“ erfassen, welche Scheidt als „Nachkommen der frühgeschichtlichen Bevölkerung des betreffenden Landesteils“ verstand (Scheidt & Wriede 1927: 121). Hatte sich Eugen Fischer noch beklagt, dass das „größte Rassenkreuzungsexperiment am Menschen“ in den USA ungenutzt abgelaufen sei, da es keine Daten hinterlassen hätte (Fischer 1913: 2), so bildeten Kirchenbücher den Ausgangspunkt für Scheidts Laboratorium der Vererbung. Sie ermöglichten die „Anwendung genetischer Fragestellungen“ (Scheidt 1929: 147). Kirchenbücher stellten – im Gegensatz zur Loseblattsammlung der physischen Anthropologie – einen bereits verschalteten Datensatz dar: sie enthielten „die Bevölkerung eines Wohngebietes in ihrem genealogischen Zusammenhang“. Die „bevölkerungsbiologische Verwertung der Kirchenbücher“ war daher, so Scheidt, die „vordringliche Aufgabe der Wissenschaft, insbesondere der Rassenbiologie“ (Scheidt 1929: 147–148).

In Form des gebundenen Buches enthielten die Kirchenbücher zwar den notwendigen Datensatz, jedoch musste dieser in ein anderes Medium übersetzt werden, um „rassenbiologisch“ auswertbar zu sein. Scheidt und seine Mitarbeiter:innen, insbesondere Irene Roethig und Wilhelm Klenck, erdachten dafür ein komplexes System der „Verkartung“. Sie überführten also die Kirchenbücher in die Form der Kartei (Scheidt 1929: 149–161). Letztere war zu Beginn der 1920er Jahre als Bürotechnik eine junge Form der Datenverarbeitung. Gab es zwar schon zuvor Zettelkästen, so entstand die Kartei in ihrer modernen Form als Bürotechnik in der zweiten Hälfte des 19. Jahrhunderts in den USA, aber erst um „1900 kam die Kartei aus Amerika wieder zurück.“ (Porstmann 1950 [1922]: 13) Der Unterschied zwischen Buch und Kartei, womit letztere den „Grundoperationen einer *Universalen Diskreten Maschine*“ genügt – nämlich „Daten zu speichern, zu prozessieren und (selbst) zu übertragen“, bestand vor allem in der Beweglichkeit ihrer Datenträger (Krajewski 2017: 10). Aus diesem dynamischen Datenspeicher, der in der Buchhaltung zur Erfassung und Kontrolle von Waren- und Kapitalströmen diente, wurden bei Scheidt „Erbströme“, die sich „rassenbiologisch“ erfassen und bevölkerungspolitisch kontrollieren ließen.

**Figure 3:**
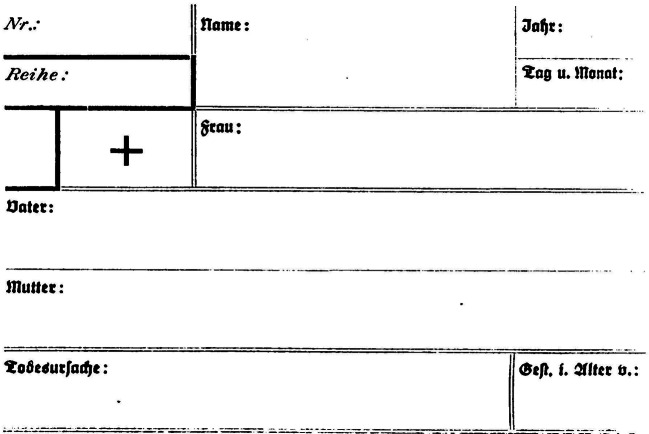

Die Praxis der „Verkartung“ oder auch „Verzettelung“ meinte zunächst, dass der gesamte Inhalt der Kirchenbücher und damit „jeder Eintrag des Taufregisters, des Sterberegisters und des Verehelichungsregisters“ auf vorgedruckte Karteikarten à 9 × 12 Zentimeter (Abb. 3) übertragen wurde (Roethig 1927: 214).

Im Fall der Finkenwerder-Studie waren dies nach Scheidts Mitarbeiterin Irene Roethig, die das im Folgenden skizzierte Verfahren schilderte, rund 25.000 Karten. Es entstanden zunächst „drei Register (in getrennten Kästen handlich aufgestellt)“, die eine erste Ordnung des Materials in alphabetischer und zeitlicher Reihenfolge zuließen. Im Anschluss daran konnte Roethig die „genealogischen Zusammenhänge“ herstellen (Roethig 1927: 215–216). In der Tat war dafür die Beweglichkeit der Datenträger zentral. Ähnlich wie die Einführung der Zählkarte im preußischen Zensus im letzten Drittel des 19. Jahrhunderts neues Wissen über die Bevölkerung produzierte (Oertzen 2017), ermöglichte die Übersetzung der Kirchenbucheinträge in Karteiform erst, die Abstammung der Bevölkerung zu erfassen. So ließen sich durch Auslegen der Karten „Geschwisterreihen“ bilden. Roethig sortierte dafür die Taufbuchkarten nach Namen und verknüpfte diese mit der Kartei der Eheschließungen, während die Kartei der Sterbedaten zur Kontrolle diente. Im Anschluss nahm Roethig die „Aufstellung der Linien“ vor – also der familiären Zusammenhänge, an deren Ende die „graphische Darstellung“ in „Form von Familientafeln“ – also Stammbäumen – stand. In diesem Prozess erhielt jede erfasste Person eine Nummer, die sowohl im Stammbaum als auch auf den zugehörigen Karteikarten eingetragen wurde. Entlang dieser Nummern, die „die ganze Bevölkerung […] durchlaufen“, sortierte Roethig die Karten erneut und führte sie in einer Kartei zusammen: „In dieser Aufarbeitung wird das Kirchenbuch nun als Grundlage für bevölkerungs- und familienbiologische Untersuchungen brauchbar sein“ (Roethig 1927: 216–219).

**Figure 4:**
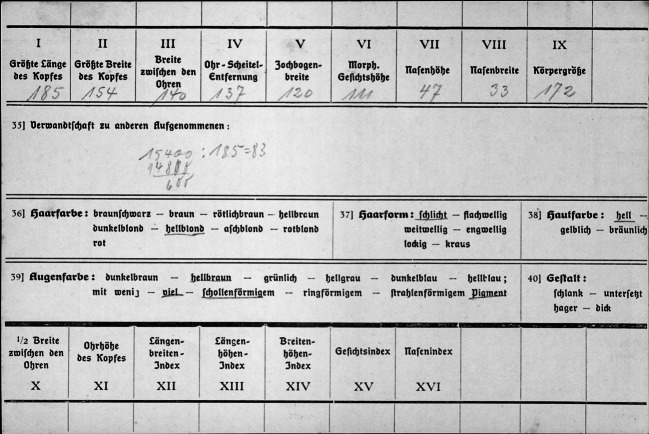

Auf diese Weise war es nach Scheidt möglich, in kurzer Zeit und mittels einer simplen Abfrage „für bestimmte Personen in der Kartei die Zusammenhänge mit anderen Personen“ festzustellen. Bei einem „nichtverarbeiteten Kirchenbuch“ war dies „natürlich nicht möglich“ (Scheidt 1929: 152). Auch Scheidts Mitarbeiter Klenck betonte, dass die Kartei den „rein zeitlich nach Art eines Tagebuchs fortgeschriebenen Inhalt der Kirchenbücher“ in eine handhab- und auswertbare Ordnung überführte (Klenck & Kopf 1938: 6). Bereits das dergestalt aufbereitete Material ließ Schlüsse einer „genetische[n] Bevölkerungsbiologie“ zu. Etwa machte es Bevölkerungsbewegungen sowie „vorhandene Unterschiede in der Stärke der Fortpflanzung“ samt Hinweisen auf deren Ursachen sichtbar (Scheidt 1929: 174). Vor allem stellten diese Datenpraktiken erst jene altansässige Bevölkerung her, anhand derer Scheidt meinte, die Herausbildung von „Rassenmerkmalen“ erfassen zu können. So ließ die genealogische Datenverarbeitung die „Träger des Volkstums“ bestimmen: Nur wer „Spuren“ – also Daten – hinterließ, wurde zum Teil des „Volkskörper[s]“ erklärt; andere Personen galten „biologisch als ‚fremde‘ [sic!]“ (Scheidt 1929: 160–161). Erst an diese Selektion schloss sich die Vermessung mittels der von Martin entwickelten Technik an. Das von Scheidt im Karteiformat entwickelte Beobachtungsblatt (Abb. 4) sah jedoch nur neun und damit deutlich weniger morphologische Merkmale vor als die großformatigen Beobachtungsblätter Martins. „Rasse“ wurde also nicht – wie in der physischen Anthropologie – allein aus morphologischen Merkmalen abgeleitet, sondern aus Abstammungsgemeinschaften.

Erfolgte die Auswertung und Verarbeitung der „so gewonnenen Beobachtungen […] nach den allgemeinen Regeln rassenkundlicher Arbeitsweise“ (Scheidt & Wriede 1927: 132) – darin der physischen Anthropologie ähnlich –, so waren die Ergebnisse doch andere. Denn anstatt, um dies in aller Kürze herunterzubrechen, aus Mittelwerten der Körpermaße ahistorische „Typen“ oder „Rassen“ zu errechnen, erkannte Scheidt eine „auslesebedingte Häufung“ (Scheidt & Wriede 1927: 121) von Merkmalen, die er zu Ergebnissen eines langen evolutionären Prozesses erklärte. Über diesen gab die als altansässig hergestellte Bevölkerung mittelbar Auskunft. Insofern fielen bei Scheidt auch der Begriff vom „Volk“ im Sinne einer Abstammungsgemeinschaft und „Rasse“ wieder stärker in eins, da die morphologische Ähnlichkeit einer Gruppe und die generationsübergreifende Erhaltung derselben letztlich „Äußerung ein und desselben Dinges, nämlich der Rasse“ seien (Scheidt 1925: 332). So meinte Scheidt anhand seiner Finkenwerder-Studie den Beweis erbracht zu haben, dass „das, was heute für die ganze Bevölkerung als typisch angesehen werden muss, durch die im Laufe der Generationen erfolgende stärkere Fortpflanzung der Bewährungstüchtigen zum ‚Typus‘ wurde.“^16^

Von Scheidts „rassenbiologischer“ Kartei im Hamburger Völkerkundemuseum, aus der obige Ergebnisse der Finkenwerder-Studie hervorgingen, ist nichts übrig geblieben. Einen mittelbaren Einblick bietet jedoch das „Archiv“, das Wilhelm Klenck während seiner von Scheidt beauftragten „rassenkundlichen“ Studie im niedersächsischen Lamstedt anlegte (Klenck 1934: 3). Offenbar ist ein Großteil jenes Datensatzes, der dem ersten Band der *Deutschen Rassenkunde* zugrunde lag (Scheidt & Klenck 1929), erhalten.^17^ Klenck imitierte die Hamburger Kartei seines Lehrers. So findet sich hier auf abertausenden Kärtchen und in einzelnen Karteikästen geordnet die Kirchenbuchverkartung für die Jahre 1647 bis 1925. Insgesamt sind darin die genealogischen Daten von über 30.000 Personen gespeichert, zudem die daraus erarbeiteten Stammtafeln sowie die Daten der anthropometrischen Aufnahmen. Die Datensätze – genealogische Kartei, Stammtafeln, Körpervermessung – sind untereinander verknüpft. Dieses Datenarrangement ermöglichte Klenck, „tief in die Vergangenheit hineinzuleuchten und Auskunft zu geben über Bevölkerungszu- und -abnahme, Ausbreitung und Aussterben von Erbstämmen, Anteil ‚fremden‘ Blutes und Grad der Inzucht, sozialen Auf- und Abstieg der Familien u. a. m.“ (Klenck 1934: 6).

Als dynamische Datenspeicher brachten Karteien jedoch nicht nur neues Wissen über „Rasse“ hervor, sondern materialisierten auch zuvor nur gedachte Kategorien und prägten die Vorstellungen von ihnen. So schlug Scheidt Anfang der 1930er Jahre vor, eine „bevölkerungsbiologische Zentralkartei des deutschen Volkes“ einzurichten. In einem „zweigeschossigen Block von 120 × 120 m“ sollten rund 600 Millionen Karteikarten gespeichert werden. In dieser Kartei wäre, so Scheidt, „der ganze Volkskörper“ vertreten und „zur Auskunft an einem Ort anwesend“ – man „könnte in 2 Stunden daran vorbeigehen“. Das Volk ließ sich über die Kartei als tatsächlich Reales imaginieren, ja die Kartei erweckte es gar zum Leben: Sie sei ein „fein und schnell reagierendes ‚Manometer‘ für die Lebenserscheinungen des Volkskörpers“. Deutlich wird dabei zugleich, dass die Kartei Volk und „Rasse“ vor allem auch dem bevölkerungspolitischen Zugriff zuführten. So wäre die erdachte Zentralkartei „für viele unerläßliche Zukunftsaufgaben der Bevölkerungspolitik und Rassenhygiene von Vorteil“ (Scheidt 1932: 565–567). Dies war auch bereits explizites Ziel der „Deutschen Rassenkunde“, die, wie Eugen Fischer schrieb, eine für „bevölkerungspolitische Verwaltungs- und Gesetzesfragen unendlich wichtige Quellensammlung“ liefere.^18^ Klenck verstand seine Datensammlung in Lamstedt als „Vorarbeit […] für bevölkerungspolitische und rassenpolitische Maßnahmen“ (Klenck 1934: 7).

Es ist somit wenig verwunderlich, dass jenes bereits in den 1920er Jahren auszumachende und vor allem auch außerhalb der Anthropologischen Abteilung des Hamburger Völkerkundemuseums gestiegene Interesse an genealogischer Datenverarbeitung in den 1930er Jahren und insbesondere ab 1933 sprunghaft zunahm (Schlumbohm 2018: 213–236; Gausemeier 1998: 82–95). Hinter diesem gestiegenen Interesse standen auch Gesetze, die der nationalsozialistische Staat mit seinen ersten Amtshandlungen erlassen hatte. Diese kodierten das Abstammungsdenken rechtlich und überführten es in eine rassistische Herrschaftspraxis – mit dem Gesetz zur Wiederherstellung des Berufsbeamtentums und dem Reichserbhofgesetz im Jahr 1933 sowie den Nürnberger Gesetzen von 1935. Ab 1933 bestand die Frage weniger darin, ob eine Totalerfassung der Bevölkerung mittels Verkartung vorgenommen werden sollte, sondern nur darin, wie dies technisch zu lösen sei. Die Kartei wurde zur rassistischen Herrschaftstechnik.

So wollte Achim Gercke (1902–1997), Sachverständiger für Rasseforschung beim Reichsministerium des Innern, „Rasse“ als „Strom des Ergbutes“ verstanden wissen. Eine „Sippenkartei für ganz Deutschland“ sollte diesen erfassen und eine „großzügige Rassenpolitik“ ermöglichen (Gercke 1933: 16–22). Kurt Mayer (1903–1945), im Jahr 1935 Nachfolger Gerckes und Leiter der nun in Reichsstelle für Sippenforschung (RfS) umbenannten Dienststelle, war pragmatischer: Er sah in der Verkartung vor allem eine Möglichkeit, den gesetzlich implementierten Abstammungsnachweis zu vereinfachen. Er hatte 1934 selbst eine Methode entwickelt, mittels welcher der Berliner Pfarrer Karl Themel die Kirchenbücher der Hauptstadt zu bevölkerungspolitischen Zwecken verkartete (Themel 1936). Ungefähr zeitgleich hatte der Reichsnährstand (RNS) in bayerischen Gemeinden nach einem anderen System zu verkarten begonnen.^19^ Mittels der Methode Demleitner-Roth – so die Namen der Erfinder – sollte der im Reichserbhofgesetz verankerte Abstammungsnachweis vereinfacht werden. Der Nationalsozialistische Lehrerbund (NSLB) sprach sich Anfang des Jahres 1937 für die Methode Wilhelm Klencks aus und wollte diese zunächst im Gau Ost-Hannover umsetzen.^20^

Insbesondere die RfS strebte Mitte der 1930er Jahre danach, die Datenerfassung zu vereinheitlichen. Während sie für die Methode Themel votierte, warb der Gauleiter Ost-Hannovers offensiv beim Reichsministerium des Innern für die Technik Klencks und Scheidts: „Da Kartei und die Sippentafeln durch ein Nummerierungsverfahren miteinander verbunden wurden, sind gesuchte Personen schnell zu finden.“ Ein Abstammungsnachweis mit 30 Ahnen ließe sich in rund einer Stunde ausstellen.^21^ Vor den Toren Hamburgs fand daraufhin im März 1937 eine Tagung statt, an der auch RfS-Leiter Mayer teilnahm. Klenck trug nicht nur seine Methode vor; er hatte auch seine „Karteien, Tafeln und Fotografien auf einen Lastwagen nach Harburg“ geschafft und dort ausgestellt.^22^ Die Methode, so hielt es das Protokoll fest, wurde als „praktisch richtig und zweckmässig“ erkannt. Wozu solche Karteien fähig seien, stellte RfS-Leiter Mayer sogleich klar: Es gelte besonders darauf zu achten, „wo Juden oder Fremdstämmige in den einzelnen Familien […] auftauchten“ – sie sollten gesondert erfasst und die Daten über Klenck an die RfS geleitet werden. In nächster Zeit würde nun die Methode Klenck auf weitere Gebiete ausgedehnt und eine „Reichsarbeitsgemeinschaft für Sippenforschung“ begründet.^23^

Zog auch Mayer seine Zustimmung zu dieser Arbeitsgemeinschaft wieder zurück, unterzeichneten „Reichsbauernführer“ Darré sowie Groß vom RPA und der NSLB-Leiter Fritz Wächtler (1891–1945) im August 1937 ein Abkommen über ihre Gründung und damit über eine Totalerfassung mittels Kirchenbuchverkartung. Das gewählte Verfahren stellte einen Kompromiss zwischen der Methode Klencks und jener von Demleitner-Roth dar. Verkartet wurde nach Klenck, die so aufbereiteten Daten wurden dann sowohl nach Klenck als auch nach der Methode Demleitner-Roth verarbeitet. Klenck publizierte gemeinsam mit Ernst Kopf vom RNS eine umfangreiche Arbeitsanweisung, die das Verfahren beschrieb. Diese „Bestandsaufnahme“, so heißt es dort, versetze in die „Lage, jede Blutslinie auf ihre Erbwertigkeit, insbesondere im Hinblick auf Ausmerzung fremden und kranken Blutes, auf Auslese und Zucht zu untersuchen“ (Klenck & Kopf 1938: 8). Über 14.000 Mitarbeiter:innen begannen 1938 im gesamten Deutschen Reich mit der Verkartung.^24^

Im Jahr 1939 waren mehr als 8,5 Millionen Karteikarten in 4000 Gemeinden ausgefüllt. Vollständig verkartet waren jedoch nur 319 Gemeinden.^25^ Durch den Beginn des Zweiten Weltkriegs kam die Arbeit bald zum Erliegen. Wie wichtig die Totalerfassung jedoch aus Sicht der NS-Rassenpolitik war, zeigt sich darin, dass 1942/1943 ein erneuter Anlauf unternommen wurde, sie umzusetzen. Dieses Mal beteiligte sich neben der in Reichssippenamt umbenannten RfS und Akteuren aus dem Umfeld des RNS auch die SS-Führung. Folgt man Wolfram Pytas Analyse dieses erneuten Vorstoßes, eine „erbbiologische Zentrale“ einzurichten, dann vermittele diese „einen beklemmenden Vorgeschmack von der nach einem ‚Endsieg‘ im NS-Rassenstaat betriebenen Politik“ (Pyta 2001: 83–85).

Die Methode, die Scheidt erstmalig bei seiner Studie auf der Elbinsel Finkenwerder erprobt hatte und die den Grundstock für seine „rassenbiologische“ Kartei im Hamburger Völkerkundemuseum bildete, erstreckte sich im Jahr 1938 auf das gesamte Deutsche Reich. Scheidts Vorstellung einer Totalerfassung von Volk und „Rasse“ war tatsächlich in die Wege geleitet worden. Offiziell spielte Scheidt dabei keine Rolle. Er galt als „weltanschaulich nicht eindeutig“ – so hatte Klenck bei einer Besprechung vernommen.^26^ Über Klenck, den Scheidt insbesondere im Frühjahr und Sommer 1937 beriet, spielte der aus ideologischen Gründen geschasste Anthropologe – aus zweiter Reihe – dennoch eine Rolle in der NS-Rassenpolitik: Er hatte eine Technik entwickelt, mittels derer Volk und „Rasse“ materialisiert und zum Gegenstand bevölkerungspolitischer Eingriffe werden konnten. Die Kartei ermöglichte als dynamischer Datenspeicher insofern nicht nur neue Praktiken der Datenspeicherung und -verarbeitung, die wiederum die Grundlage neuen Wissens und neuer Vorstellungen von Volk und „Rasse“ bildeten. Insbesondere die Beweglichkeit der Datenträger, die zudem bereits in der Kartei verschaltet waren, machte die hergestellten Differenzkategorien auch verwaltbar.

## „Rasse“ im Informationszeitalter – Rainer Knußmann und die Populationsgenetik

Im Jahr 1972 nahm der Anthropologe Rainer Knußmann den Ruf an die Universität Hamburg an und wurde Leiter des Anthropologischen Instituts. Walther Scheidt lehrte und forschte dort noch bis 1965.^27^ Knußmanns Vorgänger hatte sich jedoch spätestens in den 1940er Jahren von der „Rassenforschung“ weitestgehend ab- und psychologischer Forschung zugewandt. Mit Knußmann wurde ab den 1970er Jahren am Hamburger Institut wieder explizit „Rassenforschung“ betrieben. Erst ab Mitte der 1990er Jahre kam es zu studentischen Protesten gegen Forschung und Lehre an Knußmanns Institut. Völlig zu Recht kritisierten die Studierenden letztere als rassistisch. Sie zeigten einerseits die längeren Kontinuitäten der deutschen Anthropologie auf, in die auch Knußmann einzuordnen war. Andererseits verwiesen sie darauf, dass das, was Knußmann und seine Mitarbeiter:innen als „Rasse“ bezeichneten, als Konstrukt verstanden werden muss. Knußmann beharrte demgegenüber darauf, dass der Mensch „sehr wohl ein biologisches Wesen mit tiefer biologischer Verwurzelung“ sei und sich Menschengruppen entsprechend anhand biologistischer Differenzmarker unterscheiden ließen.^28^ Die Ergebnisse der Debatte waren vernachlässigbar; allein die Vorlesung „Rassenkunde des Menschen“, die seit den 1970er Jahren am Hamburger Institut gehalten wurde, erfuhr eine Umbenennung in „Geographische Variabilität des Menschen“.^29^ Bis auf einige Ausnahmen blieben die Inhalte jedoch weitestgehend dieselben – rassistische Inhalte, nur unter einem anderen Namen.

Jüngst haben Tino Plümecke und Katharina Schramm unterstrichen, dass einer Kritik, die „Rasse“ vornehmlich als „anachronistische Fiktion sowie als Produkt pseudowissenschaftlicher Verirrungen“ begreift, die „Bedingungen und Praktiken biologischer Wissensproduktion“ entgehen, „wodurch wichtige Aspekte des Problems Rasse aus dem Blick geraten“ (Plümecke & Schramm 2022: 179–180). Anders ausgedrückt: Die studentische Kritik an Knußmann blieb insofern unvollständig, als dass sie jene vermeintliche Natur, die Knußmann meinte zu beforschen und auf die er sich in der Debatte immer wieder bezog, nicht zum Gegenstand der Kritik machte. Wie entstand also, so muss gefragt werden, „Rasse“ in Knußmanns populationsgenetischen Studien?

Als epistemisches Erzeugnis anthropologischer Forschung hatte „Rasse“ sich im Verlauf der zweiten Hälfte des 20. Jahrhunderts gewandelt: Sie entstand nicht mehr primär über die Vermessung menschlicher Körper, sondern mittels molekulargenetischer Methoden – wurde also von äußeren Merkmalen ins Körperinnere verlagert (Reardon 2005: 45–74; Plümecke 2013). Die Molekulargenetik, so hat insbesondere Lily Kay gezeigt, entlieh ab der zweiten Hälfte des 20. Jahrhunderts ihre zentralen Metaphern dem Informationsdiskurs und war somit vor allem auf diskursiver Ebene mit den Techniken und Praktiken elektronischer Datenverarbeitung verknüpft (Kay 2001). Dieses Aufschreibesystem, so wird im Folgenden zunächst anhand der Feldforschung Knußmanns argumentiert, hatte im Hinblick auf die Konstruktion von „Rasse“ einen wirkmächtigen Effekt: Es entstand die Vorstellung, dass Menschen – oder genauer: die von ihnen entnommenen Proben – selbst als Informationsträger eingesetzt werden könnten, um auf biologisch bedingte Gruppenzugehörigkeit zu schließen. Gerade dies verschleierte wiederum, dass es nicht die unvermittelte Natur war, die die Grundlage der dabei entstehenden Differenzkategorien bildete, sondern allerlei kulturelle und politische Annahmen in die Konstruktion von „Rasse“ einflossen. Es waren also weniger Computer als datenverarbeitende Maschinen selbst, die diese Vorstellungen prägten, sondern der mit ihnen einhergehende Informationsdiskurs. Dennoch wurde „Rasse“ wiederum mittels Daten hergestellt und im Materiellen verankert.

Rainer Knußmann nahm indes eine Mittlerstellung zwischen der Anthropologie der ersten Hälfte des 20. Jahrhunderts und jener der zweiten Hälfte ein. Bevor Knußmann nach Hamburg kam, war er Schüler von Ilse Schwidetzky (1907–1997) am Anthropologischen Institut der Universität Mainz. Schwidetzky bildete wiederum als Schülerin des im Nationalsozialismus wirkmächtigen Anthropologen Egon von Eickstedt (1892–1965) ein „Scharnier“ zwischen der NS-Anthropologie und jener der Bundesrepublik (Etzemüller 2015: 56). Knußmann fand in Eickstedt somit, wie er es selbst ausdrückte, seinen „geistigen Großvater“ und forschte in dessen Tradition.^30^ Zwar grenzte Knußmann seine spätere populationsgenetische Definition von „Rasse“ von Eickstedts typologischer ab (Knußmann 1996: 407), übernahm jedoch unter anderem dessen Taxonomie sogenannter „Rassenkreise“.

Dieser Einfluss Eickstedts auf Knußmann wird auch in einer Forschungsreise sichtbar, die Knußmann im Jahr 1968 unternahm. Gemeinsam mit seiner Frau Renate brach er für vier Monate ins heutige Namibia auf, das zu dieser Zeit – und nach der deutschen Kolonialherrschaft – als Südwestafrika unter südafrikanischer Verwaltung stand. Anlass der Reise war ein von Eickstedt ausgemachtes Forschungsdesiderat um eine als „Dama“ bezeichnete Gruppe. So hatte Eickstedt allerlei Vermutungen über die Herkunft dieser Gruppe und ihre Zugehörigkeit aufgestellt, „eine eigentliche anthropologische Untersuchung fehlte“ jedoch (Knußmann & Knußmann 1969/1970: 13). Indes ist davon auszugehen, dass Knußmanns Forschungsreise auch in einem Zusammenhang mit der Apartheidpolitik Südafrikas stand: So hatte die südafrikanische Regierung wenige Jahre zuvor den sogenannten „Odendaal-Report“ veröffentlicht, der auch mittels rassistischer Segregation weiße Mehrheitsverhältnisse schaffen sollte. Für geplante Umsiedlungen und damit eine räumliche Ordnung der Bevölkerung entlang ethnopolitischer Kategorien bedurfte es entsprechenden Wissens über ebendiese. Knußmanns Kontaktmann vor Ort, Hans-Joachim Rust, arbeitete bei der S.W. A. Wissenschaftlichen Gesellschaft, die im Nachgang der deutschen Kolonialherrschaft gegründet worden war. Hatte Rust selbst Ende der 1950er Jahre eine Karte über die „Völker und Rassen“ Südwestafrikas veröffentlicht, so stand die Gesellschaft in den 1960er Jahren mit manchen Experten der Odendaal-Kommission im Austausch (Kröger 2022: 140–145).

Bei einem Blick auf die Datenproduktion während der Forschungsreise zeigt sich zwar wiederum die Mittlerstellung Knußmanns zwischen alter und neuer Anthropologie, zugleich jedoch der Siegeszug letzterer. So erhoben Rainer und Renate Knußmann sowohl morphologische Merkmale mittels Körpervermessung von 602 Personen als sie auch 408 Blutproben entnahmen (Knußmann 1969: 48). Neben einer ersten Auswertung vor Ort, bei der beide Datensätze zum Einsatz kamen, waren für die spätere Forschung zur „ungeklärte[n] Ethnogenese“^31^ der Dama, so der Forschungsbericht des Hamburger Instituts für die Jahre 1975 bis 1978, vor allem die Blutproben ausschlaggebend. Nachdem letztere in „mit Kühlaggregaten versehenen Kühltaschen“ von Windhoek nach Deutschland gebracht wurden (Knußmann 1969: 50), kamen die Proben Anfang der 1970er Jahre ans Hamburger Institut. Dort hatte Knußmann, der seine Übernahme des Instituts aus „apparativer Sicht“ als „Neugründung“ verstand, unter anderem ein serologisches Labor eingerichtet.^32^

In diesem Labor werteten die Wissenschaftler:innen um Knußmann die Proben hauptsächlich mittels Elektrophorese aus (Knußmann & Knußmann 1976; Schumacher et al. 1979). Ihrer Meinung nach ließ dies Rückschlüsse auf die „Polymorphismen des Blutes“ der Dama,^33^ also erblich bedingte Merkmale, zu. Die Elektrophorese trennt mittels elektrischer Spannung die Proteine des in ein Trägermedium gegebenen Blutes, sodass sich – wie etwa im Falle der zur Bestimmung der „Transferrintypen der Dama“ eingesetzten „Agarose-Hochspannungselektrophorese“ – ein auslesbares Muster von Banden ergab. Die darüber für 238 Proben sichtbar gemachten „Transferrintypen“ wiesen wiederum eine bestimmte Häufung auf, über die die Wissenschaftler:innen „Genfrequenzen“ errechneten. Ließen in den Augen der Forscher:innen bereits diese Genfrequenzen zu, dass die Gruppe der Dama von anderen Gruppen abgrenzbar war, so ermöglichte insbesondere ein statistisches Verfahren die Verschaltung der so gewonnenen Daten mit 53 weiteren Studien zu sogenannten afrikanischen Populationen (Schumacher et al. 1979: 101–103). Diese Studien, die unter anderem der spätere Begründer des Human Genome Diversity Project (HGDP) Luigi Cavalli-Sforza durchgeführt hatte, verweisen nun nicht nur auf die internationale Verbreitung der von Knußmann betriebenen Forschung. Vor allem entstanden Konstruktionen biologisch distinkter Menschengruppen innerhalb eines globalen Datenstroms.

Gerade dieser Datenstrom nährte die Illusion, dass „Rasse“ nun über die geeignete technische Apparatur direkt aus den Körpern beziehungsweise den ihnen entnommenen Proben auslesbar wäre. So käme die Elektrophorese – wie in einem von Knußmann herausgegebenen methodischen Handbuch zur Anthropologie zu lesen – gerade deswegen in der Populationsgenetik zum Einsatz, da sie „zur direkten Erfassung genetischer Informationen führt“ (Scheffrahn 1992: 371). Bei einem genaueren Blick auf die Praktiken der Datenproduktion während der Feldforschung Knußmanns zeigt sich jedoch, dass sich Gruppenzugehörigkeiten und Differenzkategorien gerade nicht aus jenen „Informationen“ ergaben, die sich aus den Blutproben auslesen ließen, sondern in einem höchst artifiziellen Konstruktionsprozess entstanden.

Dies wird insbesondere in der Auswahl der Proband:innen deutlich. Sie gestaltete sich, so Knußmann, als „problematisch“: Nach seiner Ansicht sollten nur „*reinblütige* Dama einbezogen werden“, doch gab darüber eben nicht das abgenommene Blut selbst Auskunft, sondern die zuvor vorgenommene Auswahl der zu kategorisierenden Menschen. Knußmann fürchtete etwa, dass er, wie er es ausdrückte, „Mischlinge“ in sein Sample miteinbezog. Sei die Zugehörigkeit einerseits nur sehr bedingt „physiognomisch erkennbar“, sollte andererseits „nicht mit einer vorgefaßten Meinung vom Aussehen der Dama an eine Probandenauswahl herangegangen werden“ – auch weil, wie Knußmann betonte, morphologische Marker kein verlässliches Kriterium der Zugehörigkeit bildeten. Für die Auswahl betrieb Knußmann „Ahnenforschung“, jedoch lagen „mangels älterer standesamtlicher Aufzeichnungen“ keine „sicheren Nachweise“ vor. Letztlich mussten sich die Wissenschaftler:innen auf mündliche Aussagen verlassen. Hier stellte sich für Knußmann jedoch ein weiteres Problem: „[D]ie Stammeszugehörigkeit ist für sie [die Dama] kein biologisches, sondern rein soziales Moment“. Anders ausgedrückt: Bildete die Gruppe in Knußmanns Blick eine biologisch distinkte Abstammungsgemeinschaft, spielte diese Kategorie in der sozialen Praxis der Gruppe keine Rolle. So hätte etwa trotz „ausführlicher Belehrung“ ein Proband darauf bestanden, dass er, obwohl sein Vater in Knußmanns Augen nicht zur Gruppe gehörte, ein „echte[r] Dama“ sei (Knußmann 1969: 46–47). Die Auswahl der Proband:innen beruhte also nicht nur auf einer höchst unsicheren Entscheidungsgrundlage, sondern stellte vor allem eine Biologisierung sozialer Kategorien dar.

Während bei der physischen Anthropologie um 1900 die mehr oder minder willkürliche Auswahl der zu vermessenden Menschen zu Widersprüchen in der Auswertung und auf längere Sicht zur Aufgabe der Annahme stabiler „Rassen“ oder auch Typen führte, hatte dies für die Konstruktion von biologisch distinkten Gruppen in populationsgenetischen Studien offenbar wenig Auswirkung. So wurden auch in anderen Studien, wie sich etwa in Diskussionen in der Vorbereitung des HGDP zeigt, die der populationsgenetischen Analyse zugrunde gelegten Gruppen durch eine Vielzahl von Differenzmarkern bestimmt – darunter vor allem kulturelle wie Sprache (Reardon 2005: 76–77). Diese der eigentlichen Datenproduktion vorgängige Konstruktion der als Populationen untersuchten Menschengruppen verschwand wiederum hinter jenen Daten, die durch technische Apparaturen wie die Elektrophorese produziert wurden. Verlagerte die Molekulargenetik „Rasse“ ins Körperinnere, so festigte sich nach und nach die Vorstellung, dass Informationen über die Zugehörigkeit menschlicher Körper in ebendiesen gespeichert seien.

Diese Vorstellung war bedingt durch zwei eng verbundene, nach dem Zweiten Weltkrieg einsetzende Prozesse: den Durchbruch der Informationstechnik sowie einen Paradigmenwechsel der Genetik. Die sich vor allem in den 1950er Jahren formierende Molekulargenetik war nicht nur materiell an neue Forschungstechnologien gebunden, welche die molekulare Struktur des Gens sichtbar machten, sondern auch symbolisch an das Aufschreibesystem elektronischer Datenverarbeitung. Sie entlehnte ihre zentralen Metaphern – Code, Programm, Information – dem Informationsdiskurs (Rheinberger & Müller-Wille 2009: 209–240; Kay 2001: 17–66). So sprach auch Knußmann von der „Erbinformation“, deren „Speicherung“ in der DNA sowie der „Mitteilung“ – also Übertragung – über die RNA; ein „genetische[r] Code“, der sich entschlüsseln ließe (Knußmann 1996: 38–43).

Auf den Einfluss von Informationstechnik und -diskurs auf „Ideologien menschlicher Verschiedenheit“ weist auch Donna Haraway hin. Dabei brachte die Übersetzung von Körpern in einen universellen Code nicht nur neue Formen der Macht – eine „Informatik der Herrschaft“ – hervor (Haraway 1995: 48–49). Sie festigte auch die hier verfolgte Vorstellung, „Rasse“ ließe sich direkt aus menschlichen Körpern auslesen, nachhaltig. Knußmann, der 1998 nicht etwa aufgrund des Protests um seine Person, sondern infolge einer Erkrankung auf eigenen Wunsch vorzeitig emeritiert wurde,^34^ war nur eine Figur jener Entwicklung, die Tino Plümecke als „Genetifizierung von Rasse“ im 20. Jahrhundert beschreibt (Plümecke 2013: 105–108). Diese Genetifizierung setzte sich indes in den 1990er Jahren vollends durch. Erst die im Humangenomprojekt vorgenommene Sequenzierung des menschlichen Genoms ermöglichte den tatsächlichen Zugriff auf die menschliche DNA. Zwischen jenen serologischen Methoden, mittels derer Knußmann über die „Polymorphismen des Blutes“ unterschiedliche Genfrequenzen sichtbar machte, und den heutzutage vorherrschenden Differenzkonstruktionen über DNA-Daten bestand jedoch „kein entscheidender Bruch“, sondern vielmehr ein „fließende[r] Übergang“ (Plümecke 2013: 185; Reardon 2005: 70–71). Ausdruck jener Genetfizierung sind nicht nur groß angelegte Forschungsvorhaben wie das HGDP in den 1990er Jahren oder die Operationalisierung dieses Wissens in Medizin und Forensik (Plümecke 2013: 193–216; Lipphardt & Niewöhner 2008). Vor allem jenseits des wissenschaftlichen Expert:innendiskurses und damit in der Rezeption molekulargenetischen Wissens lässt sich beobachten, wie obige Vorstellung weite Verbreitung findet.

So sind genetische Herkunftsanalysen für den Hausgebrauch mittlerweile weltweit zum gewinnbringenden Geschäft geworden: eine „globalisierte Konstellation von Forschung, Markt und Identitätspolitik“ (Sommer 2015: 184; Plümecke 2013: 217–223). Ein im Internet bestelltes Testkit für den Wangenabstrich wird nach Anwendung an den Anbieter geschickt, der es im Labor auswertet. Wenig später, so das Versprechen, erfahren die Kund:innen etwas über ihre „Abstammung“, „ethnische Herkunft“ oder gar die Zugehörigkeit zu einem „Urvolk“. Zwar ist dabei die Rede von „Rasse“ nur noch sehr bedingt auszumachen, so werden über das Sampling der notwendigen Referenzdatensätze für die Auswertung der Tests – darauf haben Tino Plümecke und Katharina Schramm hingewiesen – „auch Wissensgenealogien und Klassifikationen mobilisiert, die aus der Zeit rassenkundlicher Forschung stammen.“ Denn wie bei Knußmanns Feldforschung lassen sich jene Populationen, in welche die Nutzer:innen der Tests eingeschrieben werden, nicht in der unvermittelten Natur finden. Das Sampling operiert „in einer Populationslogik, die Gruppenzugehörigkeiten nicht einfach abbildet, sondern selektiv erzeugt“. Gerade dieser Prozess, auch darin den Studien Knußmanns ähnlich, geht „mit bestimmten Vorannahmen über Gruppenzugehörigkeiten“ einher (Plümecke & Schramm 2022: 188–189; Lipphardt et al. 2021). Dies bleibt der Kundschaft allerdings verborgen. So heißt es auf der der Webseite eines weltweit agierenden Anbieters solcher Tests, dass mit „innovativer Technologie“ gearbeitet würde: Nachdem sich die „biologischen Informationen“ aus der Speichelprobe auslesen und in „digitale Rohdaten“ umwandeln ließen, würden „Algorithmen“ eine „Ethnizitätsschätzung“ im Sinne der Zugehörigkeit zu biologisch distinkten Menschengruppen berechnen.^35^

Ein datengeschichtlicher Blick auf die populationsgenetischen Konstruktionen menschlicher Differenz vermag also zweierlei zu zeigen: Erstens sind die als biologisch distinkt verstandenen Populationen nicht in der Natur selbst zu finden, sondern es fließen allerlei Annahmen und unter anderem kulturelle sowie politische Differenzmarker in ihre Herstellung ein. Zweitens waren es die Praktiken und Technologien der Datenproduktion, die diesen Konstruktionsprozess wiederum verbargen. Die vor allem in ihrer symbolischen Dimension mit dem Informationsdiskurs verknüpfte Molekulargenetik brachte die Illusion hervor, dass Menschen ihre Gruppenzugehörigkeit als Informationen in sich trügen und sich diese mittels geeigneter Apparatur direkt aus den Körpern auslesen ließe.

## Eine Datengeschichte der Rassifizierung

Dieser Aufsatz lenkte den Blick auf die Aufschreibesysteme anthropologischer Forschung und folgte den von ihnen produzierten Datenströmen. Darüber konnte gezeigt werden, dass die Praktiken und Techniken der Datenproduktion, -speicherung und -zirkulation ein zentrales Moment in der Konstruktion von „Rasse“ sowie weiteren biologistischen Kategorien menschlicher Differenz waren. Nicht nur stellte die Verdatung erst her, was die Daten als unmittelbare Repräsentation der Wirklichkeit ausgaben – sie schrieben Differenzkategorien fest und operationalisierten sie für den politischen Zugriff. Zudem zeigte sich, dass die Herstellung dieser Kategorien einer jeweiligen Medientechnik aufsaß.

So ließ sich den drei betrachteten Phasen der Hamburger Anthropologie und damit den untersuchten drei Paradigmen ein jeweils spezifisches Aufschreibesystem zuordnen. Die Hamburger Südsee-Expedition stand ganz im Zeichen der physischen Anthropologie und damit der Medientechnik der Loseblattsammlung, also der Produktion von Daten mittels großformatiger individueller Erhebungsblätter. Walter Scheidt forschte in der Zwischenkriegszeit zur sogenannten „Erblehre“, deren zentrale Medientechnik die Kartei bildete. Schließlich zeigte sich anhand von Rainer Knußmann, dass die Populationsgenetik vor allem auf symbolischer Ebene mit dem Aufschreibesystem elektronischer Datenverarbeitung beziehungsweise dem Informationsdiskurs verknüpft war. Diese Medientechniken schrieben sich wiederum in die von ihnen hervorgebrachten Kategorien ein: erstens in Form statischer „Typen“ und „Rassen“ als mathematische Konstrukte einer verschalteten Datensammlung morphologischer Merkmale; zweitens in Form von Abstammungsgemeinschaften, die in Karteien als dynamischen Datenspeichern entstanden, aus denen „Rasse“ innerhalb eines nun berechenbaren „Erbstroms“ ausgelesen werden konnte sowie drittens als Vorstellung einer in den Genen codierten Information über die Zugehörigkeit zu einer Population oder auch „Rasse“.

Insbesondere im Hinblick auf die nach wie vor wirkmächtigen Konstruktionen menschlicher Differenz bestand ein Anliegen dieses Aufsatzes darin, neue Wege der Kritik der Rassifizierung auszuloten. Diese sollten sich nicht, wie in der Debatte um Rainer Knußmann geschehen, an den ontologischen Fallstricken der Frage aufreiben, ob die hier untersuchten Differenzkategorien nun in der Natur gründen oder kulturelle Produkte waren. Vielmehr, und so wird dies auch in jüngerer Zeit verstärkt vorgenommen (etwa bei Lipphardt & Niewöhner 2008; Plümecke & Schramm 2022), muss die Wissensproduktion um jene Natur wieder verstärkt ins Zentrum der Kritik gerückt werden. Eine Datengeschichte der Rassifizierung ist dabei nur einer von vielen Ansatzpunkten einer kritischen wissensgeschichtlichen Forschung. Diese vermochte jedoch nicht nur zu zeigen, dass die Natur, auf die sich Knußmann in der Debatte um das von ihm produzierte Wissen um „Rasse“ zurückzog, hochgradig artifiziell ist. Vielmehr waren es die Techniken und Praktiken der Datenverarbeitung, die eine bestimmte Vorstellung dieser angenommenen Natürlichkeit menschlicher Differenz hervorbrachten. Es wurden, so ließe sich zuspitzen, die „Einschreibungstechniken mit der Natur“ (Winthrop-Young 2005: 88) verwechselt.

## Archiv- und Sammlungsbestände

*Archiv des Altonaer Museums *(AAM)

- Nachlass Otto Lehmann, unverzeichnet

*Bördemuseum Lamstedt *(BL)

- Nachlass Wilhelm Klenck, unverzeichnet
- Material der „rassenkundlichen“ Erhebungen in Lamstedt, unverzeichnet

*Bundesarchiv Berlin-Lichterfelde *(BArch)

- R 1509, Reichssippenamt

*Hamburger Bibliothek für Universitätsgeschichte *(HBfUG)

- Ordner Biologie: Allgemein

*Museum am Rothenbaum – Kulturen und Künste der Welt, Dokumentenarchiv* (MARKK):

- 101‑1, Erste Hälfte des 20. Jahrhunderts
- SÜD, Hamburger Südsee-Expedition

*Staatsarchiv Hamburg* (StaHH)

- 361‑6, Dozenten- und Personalakten
- 354-5 I, Universität I (1919–1965)

*Universitätsarchiv Hamburg* (UaHH)

- Institut für Humanbiologie (Anthropologisches Institut), noch unverzeichnet

## Footnotes

1 Der Leserbrief, der im Folgenden zitierte Artikel sowie die weitere Presseberichterstattung finden sich in HBfUG, Ordner Biologie: Allgemein.

2 UaHH, Institut für Humanbiologie (Anthropologisches Institut), Ordner Korrespondenz Allgemein ab 1992, noch unverzeichnet, Knußmann an Ärztekammer, 01.09.1998.

3 HBfUG, Ordner Biologie: Allgemein, Arbeitsgruppe des Akademischen Senats zu Fragen der Forschung und Lehre am Institut für Humanbiologie, Bericht und Empfehlungen, 25.09.1997.

4 MARKK, SÜD 7.4.1 sowie SÜD 7.4.2, Reche, Otto. Anthropologika (Anthropol. Katalog).

5 Eine Vielzahl der ausgefüllten Beobachtungsblätter der Expedition sind archivalisch überliefert. MARKK, SÜD 7.3, Reche, Otto. Anthropologische Messblätter, sowie SÜD 2.4.1–2.4.5, Hambruch, Paul. Anthropologische Messblätter.

6 MARKK, SÜD 7.1.7, Reche, Otto. Tagebuch-Abschrift 3; MARKK, SÜD 2.1.8, Hambruch, Paul. Tagebuch, 3 Abschriften vom 21.06.1909–03.05.1910.

7 Dieses findet sich in den archivierten Messblättern Reches, MARKK, SÜD 7.3.

8 MARKK, SÜD 2.4.1, Palau. Anthrop. Aufnahmen, August 1909.

9 StaHH, 361‑6 IV 1435 Personalakte Otto Reche, Bericht über eine Dienstreise, 14.08.1913.

10 MARKK, 101‑1 Nr. 783, Anthropologische Erforschung der deutschen Bevölkerung, 02.11.1928, gez. Fischer sowie Aichel, Mollison, Reche, Saller, Scheidt, Thilenius.

11 StaHH, 364‑5 I, K 20.1.379, Anlagen I, Bericht über die rassenbiologische Forschung und den rassenbiologischen Unterricht an der Hamburgischen Universität vom 01.10.1924 bis zum 15.10.1934, 23.10.1934, gez. Scheidt.

12 StaHH, 364‑5 I, K 20.1.379 Anlagen I, Scheidt an Aufklärungsamt für Bevölkerungspolitik und Rassenpflege, Dr. Gross, 24.08.1933.

13 BL, Nachlass Wilhelm Klenck, Arbeitsgemeinschaft für Sippenforschung und Sippenpflege, 20.08.1937, gez. Gross, Darré, Wächtler, unverzeichnet.

14 AAM, Nachlass Otto Lehmann, Mappe Nr. 270, Lehmann an Scheidt, 27.02.1925; Lehmann an Wriede, 06.03.1925.

15 AAM, Nachlass Otto Lehmann, Mappe Nr. 270, Erster Tätigkeitsbereicht der rassenkundlichen Erhebungen in Deutschland, 01.08.1925, gez. Scheidt.

16 MARKK, 101‑1 Nr. 779, Vermerk vom 05.02.1926, gez. Scheidt.

17 BL, Material der „rassenkundlichen“ Erhebungen in Lamstedt, unverzeichnet.

18 MARKK, 101‑1 Nr. 783, Anthropologische Erforschung der deutschen Bevölkerung, 02.11.1928, gez. Fischer sowie Aichel, Mollison, Reche, Saller, Scheidt, Thilenius.

19 BArch, R 1509/1579, Mayer an Gauleitung Ost-Hannover der NSDAP, 12.02.1937.

20 BArch, 1509/1580, Bericht über die Tagung der Kreissachbearbeiter für Sippenkunde in Harburg, Thüringer Hof, am 05.01.1937, gez. Klenck, Bl. 90–93.

21 BArch, R 1509/1580 Gauleitung Ost-Hannover an Reichsminister des Innern, 01.03.1937, gez. Telschow, Bl. 109.

22 BL, Nachlass Wilhelm Klenck, Aus meinem Leben, undatiertes Typoskript (letzter Eintrag 1944).

23 BArch, R 1509/1580, Protokoll. Ergebnisse der sippenkundlichen Arbeitstagung des NSLB, am 13.03.1937, Bl. 152–154. Vgl. zur RAG auch Pyta (2001: 75–81).

24 BArch, R 1509/1580, Ausschnitt aus der *Frankfurter Zeitung*, Reichs-Ausgabe, 05.06.1938, Bl. 272.

25 BArch, R 1509/1580, Stand der volkstumskundlichen Arbeit am 1.1.1939 nach den bis 1.2.39 eingegangenen Berichten, Bl. 281–282.

26 BL, Nachlass Wilhelm Klenck, Klenck an Scheidt, 18.05.1937, unverzeichnet.

27 Nach seiner Emeritierung im Jahr 1963 vertrat Scheidt den Lehrstuhl für Anthropologie und leitete weiterhin das Anthropologische Institut. StaHH, 361‑6 IV 1184, Meins an Scheidt, 18.03.1964, Bl. 92.

28 HBfUG, Ordner Biologie: Allgemein, Stellungnahme Knußmanns vom 22.12.1996 zum Artikel von Volker Stahl „Streit um Schädelvermesser“ in der *Süddeutschen Zeitung* vom 07./08.12.1996.

29 HBfUG, Ordner Biologie: Allgemein, Pressestelle der Universität Hamburg, Daten und Fakten aus der Universität zum Thema „Rassenkunde“, 03.02.1997.

30 UaHH, Institut für Humanbiologie (Anthropologisches Institut), Ordner Korrespondenz Allgemein ab 1992, noch unverzeichnet, Schreiben Knußmanns, 07.07.1997.

31 UaHH, Institut für Humanbiologie (Anthropologisches Institut), Ordner Forschungsbericht + Publikationslisten, noch unverzeichnet, Forschungsbericht 1975/78, 29.06.1978.

32 HBfUG, Ordner Biologie: Allgemein, Anthropologisches Institut der Universität Hamburg – 10 Jahre seit der Wiedergründung (= 01.10.1972), Hamburg 1983, S. 3.

33 So der Titel eines zwar von Knußmann mehrfach zitierten (etwa Knußmann & Knußmann 1976: 41), jedoch offensichtlich nie erschienenen Aufsatzes.

34 StaHH, 361.6 3321, Personalakte Knußmann, Knußmann an Personalreferenten der Universität Hamburg, 15.08.1995.

35 http://www.myheritage.de/dna/dna-test-kit (20.12.2021).
